# Messenger RNA Sequencing and Pathway Analysis Provide Novel Insights Into the Susceptibility to *Salmonella enteritidis* Infection in Chickens

**DOI:** 10.3389/fgene.2018.00256

**Published:** 2018-07-13

**Authors:** Peng Li, Wenlei Fan, Nadia Everaert, Ranran Liu, Qinghe Li, Maiqing Zheng, Huanxian Cui, Guiping Zhao, Jie Wen

**Affiliations:** ^1^Institute of Animal Science, Chinese Academy of Agricultural Sciences, Beijing, China; ^2^Precision Livestock and Nutrition Unit, Gembloux Agro-Bio Tech, TERRA Teaching and Research Centre, University of Liège, Gembloux, Belgium; ^3^State Key Laboratory of Animal Nutrition, Beijing, China

**Keywords:** spleen transcriptome, *Salmonella enteritidis*, resistant and susceptible, immune-related genes and pathway, chicken

## Abstract

*Salmonella enteritidis* (SE) is a foodborne pathogen that negatively affects both animal and human health. Controlling poultry SE infection will have great practical significance for human public health, as poultry are considered to be important sources and carriers of the disease. In this study, the splenic transcriptomes of challenged-susceptible (S), challenged-resistant (R) and non-challenged (C) chicks (3-days old, specific-pathogen-free White Leghorn) were characterized in order to identify the immune-related gene markers and pathways. A total of 934 significant differentially expressed genes (DEGs) were identified in comparisons among the C, R and S birds. First reported here, the DEGs involved in the Forkhead box O (FoxO) signaling pathway, especially FoxO3, were identified as potential markers for host resistance to SE infection. The challenged-susceptible birds exhibited strong activation of the FoxO signaling pathway, which may be a major defect causing immune cell apoptosis as part of SE-induced pathology; these S birds also showed weak activation of mitogen-activated protein kinase (MAPK)-related genes, contrasting with strong splenic activation in the R birds. Interestingly, suppression of several pathways in the immune response against *Salmonella*, including cytokine-cytokine receptor interaction and Jak-STAT, was only found in S birds and there was evidence of cross-talk among these pathways, perhaps contributing to susceptibility to *Salmonella* infection. These findings will help facilitate understanding resistance and susceptibility to SE infection in the earliest phases of the host immune response through *Salmonella*-induced pathways, provide new approaches to develop strategies for SE prevention and treatment, and may enhance innate resistance by genetic selection in animals.

## Background

*Salmonella enteritidis* (SE) is an enteric bacterium that can colonize chickens, contaminating meat and eggs; it does not cause production losses, but birds carry a bacterial burden with non-obvious symptoms, thereby constituting an insidious risk for public health (Barrow et al., 2012; Calenge and Beaumont, 2012; Kogut and Arsenault, 2017). SE is among the top ranking food-borne pathogens causing huge economic and human life losses. Poultry are considered to be important sources and carriers of the disease. Although use of appropriate control measures can reduce *Salmonella* contamination in poultry, *Salmonella* cases continue (Inns et al., 2015). Control of SE, therefore, is highly desirable from the perspective of both animal and human health. In recent years, genetic selection of birds is considered to be an efficient and permanent way to control *Salmonella* infection (Berthelot et al., 1998; Kaiser and Lamont, 2001; Gou et al., 2012; Kogut et al., 2012; Li et al., 2017b). A better understanding of host immunological response mechanisms should be given priority in achieving this goal.

The main route of SE infection is the oral intake of contaminated feed or water. From the intestinal tract, SE can quickly enter the bloodstream and colonize the internal organs including liver, spleen and heart (Chappell et al., 2009). The spleen plays a major role in detecting cell damage during bacterial infection and in the pathogenic mechanisms of bacterial clearance (Altamura et al., 2001). Increasing evidence suggests that the spleen plays a greater role in immune function in avian than in mammalian species, and is responsible for an immediate immune reaction after recognizing pathogens by filtering antigens from the blood (Smith and Hunt, 2004; Li et al., 2017a).

Although there have been several previous studies focusing on the splenic transcriptome following infection with *Salmonella enterica* (Zhou and Lamont, 2007; Matulova et al., 2012), avian pathogenic *Escherichia coli* (APEC) (Sandford et al., 2011; Nie et al., 2012) and virus (Wang et al., 2006; Haq et al., 2010; Smith et al., 2011), little is known about immune-related genes and pathways between resistant and susceptible birds during the course of SE pathogenesis. This paper identifies genes and pathways that are differently expressed in susceptible versus resistant chickens, after challenge with SE, to aid understanding of host immune resistance to SE infection; an earlier report (Li et al., 2017a) presented differences that were apparent at the miRNA level.

## Materials and Methods

### Ethics Statement

All animal care and experimental procedures were approved by the Institute of Animal Sciences, Chinese Academy of Agricultural Sciences (approval number: IASCAAS-AE20140615).

### Animals and Sample Collection

Specific-pathogen-free chicks (White Leghorn) were obtained from the Beijing Laboratory Animal Research Center and were treated as described earlier (Gou et al., 2012; Li et al., 2017a). Groups of 30 SE-challenged chicks were initially screened at 0.5, 1, 2, 4, 6, and 8-days post infection at 3 days of age; 24-h post infection was found to be optimal for showing differences (clinical symptoms and bacterial burden) between the 3 groups to best expose potential differences in mRNA expression. The challenged-susceptible (S) chicks exhibited severe clinical symptoms (diarrhea, drooping wings and dying) and higher bacterial loads (>10^7^ cfu/10 μL blood) compared with the others. Chicks with only slight clinical symptoms and lower bacterial loads (<10^5^ cfu/10 μL blood) were identified as challenged-resistant (R) birds. Six challenged chickens conforming to the requirements (3 R and 3 S) were selected from the 30 chickens sampled at 24 h. No Salmonella were detected in the PBS-challenged chicks and 3 were randomly chosen from 15 chicks as controls (C) at same time-point. As shown in Supplementary Figure S1, the number of SE (log_10_ cfu) measured in blood or spleen were closely related (*R*^2^ = 0.892, *n* = 30). The bacterial burden in blood of S chicks exceeded that in R chicks (Supplementary Figure S2, *P* < 0.01).

### RNA Extraction, cDNA Library Preparation, and RNA Sequencing

Total splenic RNA was extracted from each of the 9 birds, using RNeasy Plus Micro Kit (74034) (Qiagen, Hilden, Germany) following the manufacturer’s protocol. The total RNA quantity was evaluated using Bioanalyzer 2100 and RNA Integrity Number (RIN) scores exceeding 8.0. For each sample, approximately 3 μg of total RNA was depleted of ribosomal RNA (Epicentre Ribo-Zero Gold Kit, Illumina, San Diego, CA, United States). Following purification, the RNA fractions were broken into small pieces using divalent cations at high temperature. And the final cDNA library was generated using reverse transcription amplification of cleaved RNA fragments. Sample Preparation Kit (Illumina, San Diego, CA, United States), and paired-end sequencing was performed on an Illumina Hiseq2000 by LC-BIO (LC Sciences, Houston, TX, United States) and 100 bp paired-end reads were generated. Quality control of reads was determined by FastQC software (v0.10.1), details of which were described earlier (Li et al., 2017a). In brief, clear data were obtained from the raw reads, eliminating contamination with sequencing adapters or poly-N and low quality reads (*Q* values < 20), along with potential residual ribosome RNA. Clean reads were aligned to the reference genome (Gallus gallus 4.0) database using TopHat (Trapnell et al., 2009) software (v2.0.9) and Bowtie (Langmead and Salzberg, 2012) (v2.0.0), and the mapped transcripts were assembled *de novo* using Cufflinks (Trapnell et al., 2010). The RNA-seq data can be obtained from the BIG Data Center Members (2017) database with the accession number CRA000463.

### Differentially Expressed Genes and Function Enrichment Analysis

Fragments per kilobase of exon per million mapped reads (FPKM) was used to quantify the abundance of mRNAs using the Cufflinks package (v2.1.1). Analysis of DEGs between the 3 groups of chickens was performed with a false discovery rate (FDR < 0.1), *P* < 0.05, and |fold change| >1.5. The DEGs were used to implement GO and Kyoto encyclopedia of genes and genomes (KEGG) pathway analysis using KOBAS (Xie et al., 2011) (v3.0) and DAVID (Dennis et al., 2003) (v6.8). Volcano plots were performed using OmicShare tools^1^. The normalized read counts of some mRNAs were set to be 0.01 for further calculation if they had no reads in the library.

### Validation of DE Genes by Quantitative Real-Time PCR (qPCR)

To validate the DEGs identified by RNA-seq, qPCR analyses were performed to measure transcript abundance of 16 selected genes (*IL10RB*, *TNFSF10*, *LAMP1*, *ZNF207*, *CCND1*, *GJA1*, *FTH*, *HBBA*, *GAL1*, *CREBBP*, *BRI3BP*, *SOCS1*, *ICOS*, *CTLA4*, *AVD*, and *IL8*) in an ABI 7500 Detection System (Applied Biosystems, Foster, CA, United States). The candidate genes were selected for their involvement in multiple immune response pathways and their levels of differential expression (high, mean FPKM > 1000; middle, 70 < mean FPKM < 200; low, mean FPKM < 50) in the RNA-seq analysis. cDNA was obtained from the same individual samples used in RNA-seq. The qPCR amplification was as follows: each qPCR reaction (20 μl), run in triplicate, consisted of either 1 μl of template cDNA, 10 μl of 2 × KAPA SYBR FAST qPCR Master Mix (Roche, Shanghai, PRC), 0.4 μl ROX Low, 0.5 μl of each primers, and 7.6 μl PCR-grade water. The qPCR program was performed following the instructions of ABI 7500 with default parameters. 2^-ΔΔCt^ method (Livak and Schmittgen, 2001) was used to calculate the relative mRNA abundance. β-actin was used as the housekeeping gene and all primers of examined genes are described in Supplementary Table S5. Three independent replications were used for each assay and data are presented as means ± SD. Student’s *t*-test was used to compare the different expression of genes in each comparison and *P* < 0.05 was considered to be statistically significant.

## Results

### Sequencing of Splenic Transcriptomes

**FIGURE 1:**
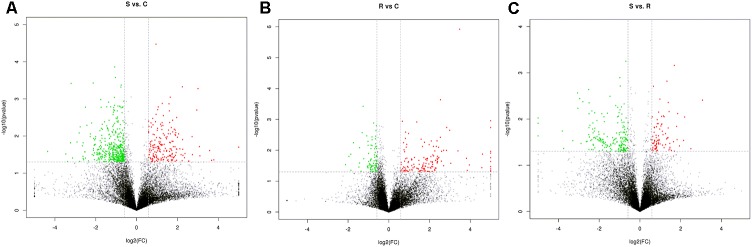
Identification of genes differentially expressed among S, R, and C chickens during SE infection. Volcano plot showing differentially expressed genes (DEGs) S vs. C **(A)**, S vs. R **(B)**, and R vs. C **(C)** after *Salmonella* infection. S, challenged-susceptible; R, challenged-resistant; C, non-challenged controls.

Next generation sequencing of splenic samples collected at 24-h post-infection produced minimum amount of 11G raw data for each of the 9 libraries. Around 95% of the clean reads had quality scores exceeding the *Q* 20 value. After removing the interference data, an average of 71.4% high quality reads was mapped to the chicken reference genome Gallus gallus 4 (see Supplementary Table S1). Among the total of 15,278 detected genes, 1,666 were novel and 11,169 genes were considered in further statistical analysis. Volcano plots, integrating both the *P*-value and fold-change of each transcript were constructed, to show the general scattering of the transcripts and filter the DEGs between the S vs. C, R vs. C and S vs. R, comparisons (**Figures 1A–C**).

### Differential Expression of mRNAs in Response to *Salmonella* Infection

**FIGURE 2:**
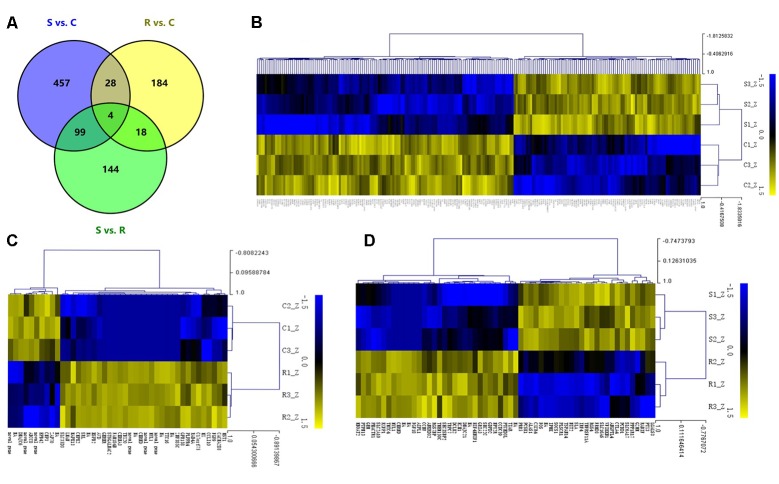
Different expression profiles of unique mRNAs in host immune response to SE infection. **(A)** Venn diagram shows the overlap of DEGs among the 3 groups; numbers are the DEGs in each comparison. **(B–D)** The heat map of unique DEGs in S vs. C, R vs. C, and S vs. R, respectively (FC > 2 and FDR < 0.05). S, challenged-susceptible; R, challenged-resistant; C, non-challenged controls.

The DEGs in spleens of the controls, resistant and susceptible birds were examined. A total of 934 significant DEGs were identified among the S, R and C chickens (**Figure 2A**). As shown in **Figure 2**, 588 genes differed between S and C (176 up- and 412 down-regulated); for R vs. C, 234 differed (145 up- and 89 down-regulated); and 265 genes (80 up- and 185 down-regulated) were DEGs between S and R birds (Supplementary Tables S2–S4). A total of 32 DEGs were shared in comparisons between S vs. C and R vs. C birds while 144 DEGs were uniquely expressed in the S vs. R comparison. Only 4 DEGs were co-expressed in the 3 contrasts. Hierarchical clustering analysis of the DEGs in each comparison demonstrated that it was appropriate to classify the challenged birds as being resistant or susceptible from their phenotypic evaluation (**Figures 2B–D**). Several immune-related genes such as *SOCS1*, *CXCR4* and *FOS* were significantly up-regulated (*P* < 0.01) after challenge with *Salmonella* (log2 FC 3.06, 3.69, and 2.50, respectively).

### Significant GO Terms and KEGG Analysis

All the DEGs and unique DEGs in each comparison among S, R, and C birds were analyzed using GO and KEGG enrichment. In this study, potential function analysis of all 934 DEGs showed that some significantly enriched GO Terms were mainly involved in channel activity and transmembrane transport but several top immune-related terms were also enriched (*P* < 0.05), including regulation of toll-like receptor signaling pathway (GO:0034121), immune response-activating signal transduction (GO:0002757), B cell receptor signaling pathway (GO:0050853) and regulation of response to stimulus (GO:0048583) (**Table 1**). In addition, Neuroactive ligand-receptor interaction, Cytokine-cytokine receptor interaction and FoxO signaling pathway were significantly changed in response to SE infection (*P* < 0.03, **Table 2**).

**Table 1:** Immune-related biological processes identified by gene ontology analysis of differentially expressed genes.

Term	Description	Count	*P*-Value
GO:0015267	Channel activity	17	0.004
GO:0022803	Passive transmembrane transporter activity	17	0.004
GO:0005216	Ion channel activity	15	0.011
GO:0038023	Signaling receptor activity	27	0.017
GO:0004872	Receptor activity	30	0.019
GO:0002224	Toll-like receptor signaling pathway	5	0.021
GO:0050853	B cell receptor signaling pathway	3	0.022
GO:0048583	Regulation of response to stimulus	59	0.031
GO:0002253	Activation of immune response	8	0.036
GO:0050778	Positive regulation of immune response	10	0.040
GO:0043065	Positive regulation of apoptotic process	12	0.047
GO:0045088	Regulation of innate immune response	6	0.048


*All DEGs among C, R, and S chickens were used to identify enriched biological functions (*P*-value < 0.05)*.

**Table 2:** Significantly changed immune-related pathways in different contrasts.

Class	Term	Count	Percents (%)	*P*-value	Fold enrichment
All DEGs	Neuroactive ligand-receptor interaction	27	3.08	0.006	1.73
	Cytokine-cytokine receptor interaction	18	2.05	0.018	1.83
	FoxO signaling pathway	14	1.60	0.025	1.95
	MAPK signaling pathway	21	2.39	0.027	1.65
S vs. C	Cytokine-cytokine receptor interaction	16	2.86	0.001	2.53
	FoxO signaling pathway	11	1.96	0.015	2.39
	Jak-STAT signaling pathway	11	1.96	0.012	2.49
R vs. C	Neuroactive ligand-receptor interaction	13	6.22	0.000	3.48
	MAPK signaling pathway	8	3.83	0.028	2.64
S vs. R	Neuroactive ligand-receptor interaction	10	4.24	0.025	2.31
	Cytokine-cytokine receptor interaction	7	2.97	0.051	2.56

*KEGG pathway analysis was performed with DAVID. *P*-value < 0.05 was considered to be statistically significant pathway*.

Potential functional analyses for host immune responses to SE infection between S and R chickens were further characterized. In the S vs. R comparisons, Neuroactive ligand-receptor interaction and Cytokine-cytokine receptor interaction pathway were enriched (*P* < 0.05) (**Table 2**); the top 3 enriched GO terms were intracellular ligand-gated ion channel activity, ligand-gated channel activity and ligand-gated ion channel activity (*P* < 0.01). For S vs. C, 3 pathways were enriched (*P* < 0.05), viz. Cytokine-cytokine receptor interaction, FoxO signaling pathway and Jak-STAT signaling pathway (**Table 2**); and several top GO terms of the immune response were enriched (*P* < 0.01), including B cell receptor signaling pathway (GO:0050853), regulation of response to stimulus (GO:0048583), and immune response-regulating signaling pathway (GO:0002764). In the R vs. C comparisons, Neuroactive ligand-receptor interaction and MAPK signaling pathway were enriched (*P* < 0.05) (**Table 2**); the enriched GO terms (*P* < 0.01) were mainly involved in channel activity, transmembrane transport, cardiac muscle cell proliferation and receptor activity, such as cation channel activity (GO:0005261), transmembrane transporter complex (GO:1902495), signaling receptor activity (GO:0038023) and oxygen transport (GO:0015671). These results indicate that compared to resistant birds, susceptible birds extensively initiate their pathways of immune response, signal transduction, and signal molecules and interaction, presumably in an attempt to resist SE infection.

### Quantitative Real-Time PCR (qPCR) Validation

**FIGURE 3:**
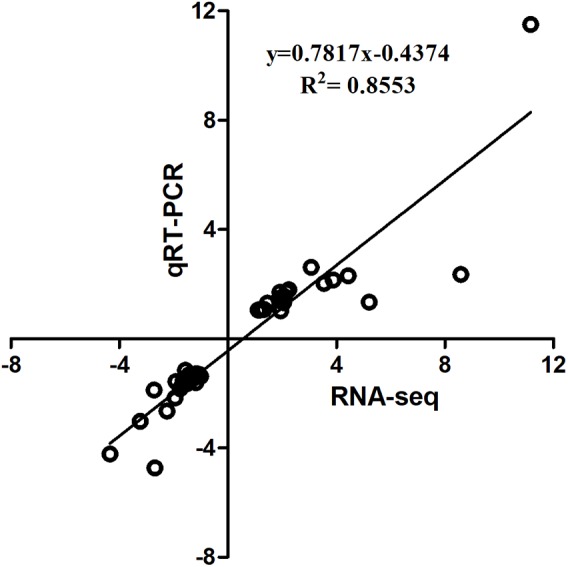
Linear regression fitted for Log_2_ Fold Change (FC) of selected genes determined via qPCR and RNA-seq. The selected genes in each comparison were used for linear regression analysis. Log_2_ FC in RNA-seq equals 2^-ΔΔCt^ in qPCR for each comparison.

The qPCR assays were conducted to validate 16 selected DEGs from RNA-seq: *IL10RB*, *TNFSF10*, *LAMP1*, *ZNF207*, *CCND1*, *GJA1*, *FTH*, *HBBA*, *GAL1*, *CREBBP*, *BRI3BP*, *SOCS1*, *ICOS*, *CTLA4*, *AVD*, and *IL8*. Pearson’s correlation of the fold-changes between qPCR and RNA-seq was 0.92 (**Figure 3**). Overall, the RNA-seq results were considered to be reliable and appropriate for further analysis.

## Discussion

Although there are previous studies focused on the chicken splenic transcriptome following *Salmonella* challenge, the novel experimental design of the current study enabled exposure of the resistance and susceptibility mechanisms of phenotypically different birds in host immune response to *Salmonella enteritidis* infection. In this research, both the extent of clinical symptoms and the bacterial load in blood were used to assess birds after infection to distinguish resistant from susceptible chickens. And RNA-seq was used here to identify differences splenic mRNAs expression profiles in chicks following SE infection. A total of 934 DEGs were identified among the C, R, and S birds. After SE infection, several up-regulated unique DEGs were mainly related to immune function, such as *FOS*, *SOCS1*, *IL-18*, *IKBKB*, *CXCR4*, *CTAL4*, *IL10RA*, *IL10RB*, *IL1RAP*, and *AVD*. These findings differ from the splenic results after *Salmonella* infection (Matulova et al., 2012), although they did identify *AVD* and immune responsive gene 1 (*IRG1*). The differences may have arisen from genetics or ages of the chickens used, or the bacterium used for challenge (Zekarias et al., 2002; Beaumont et al., 2009; Redmond et al., 2009). In generation of the heatmaps, genes included were largely driven by the S chicks. Comparing the change for each of the 3 groups, they clustered as expected based on earlier contrast comparisons (**Figure 2**).

It was clear, from the bacterial burden in blood, that septic infection occurred in the challenged birds. In response to systemic infection with *S. typhimurium*, pro-inflammatory cytokines that are host-produced are critical for the control of bacterial growth but bacterial clearance is dependent on the successful activation of CD4+ T cells, especially in peripheral immune organs (Talbot et al., 2009). Unfortunately, high doses of LPS or *Salmonella* can result in production of excess amounts of pro-inflammatory cytokines, or a “cytokine storm,” leading to endotoxin shock or sepsis-related deaths (Cohen, 2002; Clark and Vissel, 2017; Netea et al., 2017). Thus, the potential influence of over-expression of inflammatory cytokines due to hypersensitivity response to SE in susceptible birds was also considered in this study.

After challenge with SE, the significantly changed pathways included Cytokine-cytokine receptor interaction, FoxO signaling pathway, Neuroactive ligand-receptor interaction and MAPK signaling pathway. Consistent with previous studies (Chiang et al., 2008; Matulova et al., 2012; Li et al., 2017a), many immune-related pathways (Cytokine-cytokine receptor interaction, MAPK, and Jak-STAT signaling pathway) have been identified in susceptible chickens following *Salmonella* infection. Importantly, the FoxO signaling pathway is reported here for the first time.

**FIGURE 4:**
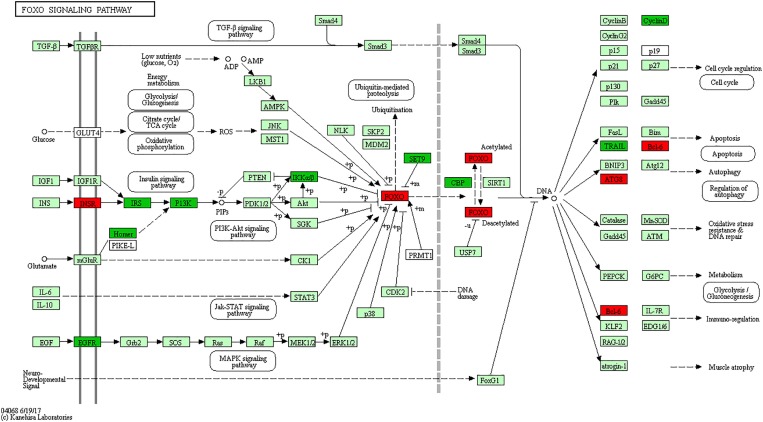
FoxO signaling pathway. Red, significantly up-regulated differentially expressed genes. Green, significantly down-regulated differentially expressed genes. Light green represents the genes involved in the pathway.

The Forkhead box O (FOXO) is one subfamily of the fork head transcription factor family with important roles in cell fate decisions, including cellular differentiation, apoptosis, cell proliferation, DNA damage and repair and as mediators of oxidative stress (Vurusaner et al., 2012; Farhan et al., 2017). FoxO activity is mainly regulated by the PI3K (phosphoinositide 3-kinase) pathway, whereas FoxO function is negatively “fine-tuned” by protein kinase B (PKB; also known as AKT), casein kinase 1 (CK1) and IκB kinase (IKK) (Peng, 2008). Research shows that *FoxO3* gene is strongly considered to regulate lymphoid homeostasis in host immune system (Lin et al., 2004; Lu et al., 2017). For instance, *FoxO3a* overexpression induces apoptosis in a human leukemia T cell line (Brunet et al., 1999), murine CTLL-2 T cell line (Stahl et al., 2002), murine pre-B cell line Ba/F3 (Dijkers et al., 2002), murine peritoneal macrophages (Senokuchi et al., 2008) and BCG-infected macrophages (Haoues et al., 2014). Deficiency of *FoxO3a* in mice leads to spontaneous, autoreactive helper T cell activation and Th1 and Th2 cytokine production (Peng, 2008), which is required for controlling bacterial growth and clearance following *Salmonella* infection (Chappell et al., 2009; Talbot et al., 2009). In addition, Foxo3a clearly plays critical roles in neutrophil survival, as demonstrated by Foxo3a-deficient mice which are resistant to both peritonitis and arthritis (Jonsson et al., 2005). In the current study, signaling adapter molecules *EGFR*, *IRS1* and *PIK3CD* of the PI3K pathway, as well as *IKBKB* gene of IκB kinase, were all significantly reduced in S vs. C birds. Of particular note, the gene *FoxO3* and apoptosis gene *Bcl-6* were significantly up-regulated only in susceptible (S) birds (**Figure 4**). These results, together with what is known of the immunobiology of avian systemic salmonellosis, indicate that the FoxO signaling pathway plays an important role in response against SE infection. Based on the above analysis, it is hypothesized that the hyperactive *FoxO3* in susceptible chickens might both enhance apoptosis of T, and B lymphocytes and macrophages in the spleen, and constrain production of Th1 and Th2 cytokine, all of which are necessary for immunological clearance during the early stage of *Salmonella* infection.

**FIGURE 5:**
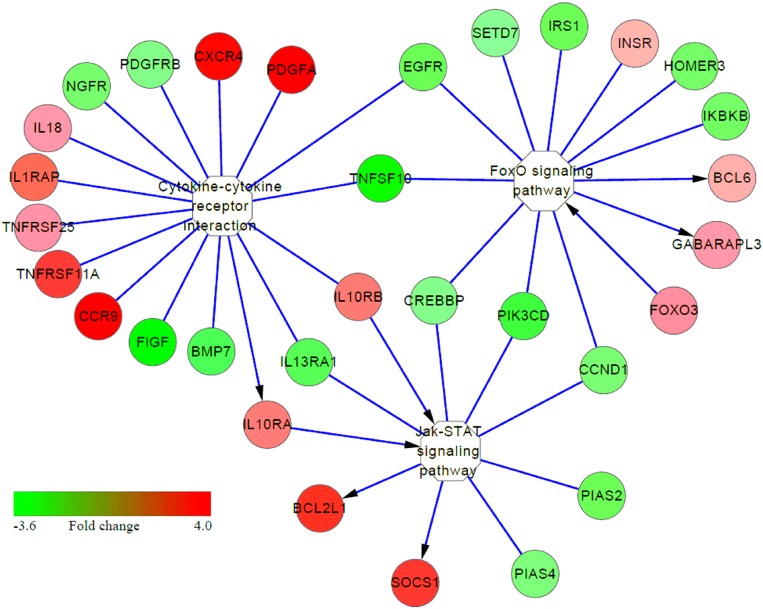
Interaction among several signaling pathways involved in susceptibility to *Salmonella enteritidis* infection. Color column from red to green represents the fold change (FC 4.0 to –3.6) of genes in S vs. C or S vs. R chickens. Red represents up-regulation and green represents down-regulation; yellow represents the pathway. The arrow represents direct activation.

Many immune-related pathways were significantly induced in S birds (S vs. C or S vs. R comparisons), including cytokine-cytokine receptor interaction, Jak-STAT, MAPK signaling pathway and neuroactive ligand-receptor interaction (**Table 2**). Interestingly, cytokine-cytokine receptor interaction, MAPK, and Jak-STAT signaling had cross-talk with activating the FoxO signaling pathway (**Figure 5**). These results suggested that multiple signaling pathway cascades control *Salmonella* invasion and clearance. In addition, increased expression of many genes in these identified pathways, in response to APEC infection, have also been demonstrated (Li et al., 2011; Sandford et al., 2012; Sun et al., 2015, 2016).

*CXCR4* is expressed in multifarious types of cancer. This cytokine receptor and its ligand are also involved in the recruitment of T-cells at the site of the immune or inflammatory reactions (Yusuf et al., 2005; Sallusto and Baggiolini, 2008). TNFRSF11A is a member of the tumor necrosis factor receptor (TNFR) molecular sub-family, also known as receptor activator of nuclear factor κB (RANK). Most commonly, over-expression of RANK alone is sufficient to activate the NF-κB pathway (Tracey et al., 2008). Interleukin 10 receptor A and B (*IL10RA*, and *IL10RB*) are expressed in most immune cells, and very low expression levels have been observed on a variety of non-hematopoietic cells (Moore et al., 2001; Yao et al., 2012). Differential expression of *IL-10RA* plays an important role in IL-10-mediated immune regulation, and activation of monocytes and neutrophils increases mRNA expression, whereas expression levels have been shown to decrease following stimulation of human T-cells, B-cells and NK cells (Carson et al., 1995; Jurlander et al., 1997; Denning et al., 2000; Yao et al., 2012). IL-1 receptor accessory protein (*IL1RAP*) mediates the response to IL-1, IL-33, and IL-36 and has been shown to regulate the inflammatory response, as well as activation of T lymphocytes and mast cells (Barreyro et al., 2012; Boraschi and Tagliabue, 2013; Dinarello, 2018). High *IL1RAP* expression is associated with poor overall survival in acute myeloid leukemia (AML) patients (Barreyro et al., 2012); although several receptors genes were increased here, only IL18 genes were significantly up-regulated. The expression of *IL18* was lower in tumor-associated macrophages cultured with metastatic gastric cancer cell lines (Shen et al., 2012). In the current study, the key genes (*CXCR4*, *TNFRSF11A*, *IL10RAP*, and *IL10RB*) in the cytokine-cytokine receptor interaction pathway had increased expression, both in S vs. C and S vs. R comparisons. While the inflammatory cytokines response is critical for the control of bacterial growth (Cross et al., 1995), excessive cytokines production can lead to endotoxic shock or sepsis-related deaths (Cohen, 2002; Clark and Vissel, 2017; Netea et al., 2017). Overall, these results suggest that susceptible birds showed hypersensitivity to acute SE infections and that the cytokine-cytokine receptor interaction pathway is an important mediator in SE-induced pathogenesis.

The Jak-STAT pathway is needful to ensure T and B-cell development (Rawlings et al., 2004). In the present study, various genes involved in the Jak-STAT signaling pathway had increased expression in susceptible birds than in non-infected controls, including *IL10RA*, *IL10RB*, BCL2-like 1 (*BCL2L1*) and suppressor of cytokine signaling 1 (*SOCS1*). BCL2L1 is one the family of Bcl-2 proteins with important roles in the regulation of mitochondrial pathway of apoptosis (Cory and Adams, 2002) and *SOCS1* is a negative regulator of LPS-induced macrophage activation (Kinjyo et al., 2002; Alvarez et al., 2017). Interestingly, three overlapping elements were found in Jak-STAT and cytokine-cytokine receptor interaction pathways in susceptible birds, both in S vs. C and S vs. R comparisons (**Figure 5**). These results indicate that susceptible birds extensively activate key pathways of immune response, signal transduction, and signal molecules and interaction in an attempt to resist SE infection, but fail to do so and succumb.

In addition, resistant chicks seem to activate the MAPK signaling in regulating the host response to SE infection. MAPK signaling was shown to be activated in chicks when pathogenic bacteria invaded (Withanage et al., 2004). It was reported that P38 MAPK is very important for B-cell development and is a survival mediator for T-cells in human inflamed tissues (Huang et al., 2009). In the current study, 5 up-regulated DE genes (*MAPT*, *MAPK13*, *CACNA2D3*, *CACNG5* and *FGF9*) participated in activating MAPK signaling pathway in resistant birds (Supplementary Table S3). These results are consistent with SE-infected, resistant birds increasing proliferation of T- and B- lymphocytes in the spleen to achieve protection against *Salmonella*.

## Conclusion

The current study is the first to characterize the splenic transcriptomes of 3 categories of chicks in response to infection with *Salmonella enteritidis*; challenged birds that were resistant (R), those that succumbed clinically (S) and non-challenged controls (C). A total of 934 DEGs were identified in comparisons between the C, R and S birds (R vs. C, S vs. C, and S vs. R). The DE genes involved in the FoxO signaling pathway, especially FoxO3, is reported here for the first time, and is identified as a potential marker of host resistance to SE infection. There was strong activation of the FoxO signaling pathway in S birds, which may be a major defect causing intracellular apoptosis of immune cells as part of SE-induced pathologenesis. Challenged-susceptible birds failed to activate components of the MAPK-related pathway in spleen whereas it was strongly activated in the R birds. Although cytokine-cytokine receptor interaction and Jak-STAT signaling pathway were activated extensively and there was cross-talk between them in challenged-susceptible birds, their role in negatively regulating the immune response against *Salmonella* likely aggravated the bacterial infections. However, the chicks used here had a consistent genetic background (White Leghorn), health status and management, a potential confounding genetic effect such as population stratification may be present, possibly affecting the results. This may influence the screening of candidate genes using randomly selected control chicks. Further analysis using birds where this possibility is eliminated is warranted. These findings will facilitate the understanding of resistance and susceptibility to SE infection in the earliest phases of the host immune response and provides new approaches to developing strategies for SE prevention and treatment.

## Author Contributions

PL and WF performed the experiments and data analysis and draft writing. NE contributed to experimental design and revised the manuscript. RL, QL, MZ, and HC contributed to animal experiments and data analysis. GZ and JW helped design the study and supervised and coordinated the study. All authors reviewed the manuscript.

## Conflict of Interest Statement

The authors declare that the research was conducted in the absence of any commercial or financial relationships that could be construed as a potential conflict of interest. The reviewer CH and handling Editor declared their shared affiliation.

## Footnotes

1 www.omicshare.com/tools

## Abbreviations

AVD: avidin
BCL2L1: B-cell lymphoma-2 like 1
Bcl-6: B-cell CLL/lymphoma 6
BRI3BP: brain protein I3-binding protein
CACNA2D3: calcium voltage-gated channel auxiliary subunit alpha 2 delta 3
CACNG5: calcium voltage-gated channel auxiliary subunit gamma 5
CCND1: cyclin D1
CREB: cAMP responsive element-binding protein
CREBBP: CREB binding protein
CTLA4: cytotoxic T-lymphocyte associated protein 4
CXCR4: C-X-C motif chemokine receptor 4
DEGs: differentially expressed genes
EGFR: epidermal growth factor receptor
FDR: false discovery rate
FGF9: fibroblast growth factor 9
FOS: Fos proto-oncogene AP-1 transcription factor subunit
FoxO: Forkhead box-O transcription factor
FTH: ferritin heavy chain 1
GAL1: Galactokinase 1, also known as AvBD1, avian beta-defensin 1
GJA1: gap junction protein alpha 1
GO: Gene ontology
HBBA: Hemoglobin beta, subunit A
ICOS: Inducible T-cell costimulator
IKBKB: Inhibitor of nuclear factor kappa B kinase subunit beta
IL: Interleukin
IL10RA/B: Interleukin 10 receptor subunit alpha/beta
IL1RAP: Interleukin 1 receptor accessory protein
IRS1: Insulin receptor substrate 1
Jak-STAT: Janus kinase-signal transducer and activator of transcription
KEGG: Kyoto encyclopedia of genes and genomes
LAMP1: lysosomal associated membrane protein 1
MAPK: mitogen-activated protein kinase
MAPT: Microtubule associated protein tau
PIK3CD: Phosphatidylinositol-4,5-bisphosphate 3-kinase catalytic subunit delta
SE: *Salmonella enteritidis;*
SOCS1: Suppressor of cytokine signaling 1
Th: helper T cell
TNFRSF11A: TNF receptor superfamily member 11a
TNFSF10: tumor necrosis factor superfamily member 10
ZNF207: zinc finger protein 207
