# Supplementary material for: Messenger RNA Sequencing and Pathway Analysis Provide Novel Insights Into the Susceptibility to Salmonella enteritidis Infection in Chickens
Source: Front Genet. 2018 Jul 13;9:256. doi: 10.3389/fgene.2018.00256 (PMC6055056; doi:10.3389/fgene.2018.00256)
Supplement: Supplementary file 2 [file Data_Sheet_1.docx]

Supplementary Material

**Messenger RNA sequencing and pathway analysis provide novel insights into the susceptibility to** ***Salmonella enteritidis* infection in chickens**

Peng Li, Wenlei Fan, Nadia Everaert, Ranran Liu, Qinghe Li, Maiqing Zheng, Huanxian Cui, Guiping Zhao*, Jie Wen*

*** Correspondence:** Corresponding Author: [zhaoguiping@caas.cn](mailto:zhaoguiping@caas.cn) and [wenjie@caas.cn](mailto:wenjie@caas.cn)

**Supplementary Information**

**Figure S1. Analysis of the correlation between the amount of bacterial burden in peripheral blood and spleen tissue.** Data are presented as the bacterial loads of five chickens on 0.5, 1, 2, 4, 6 and 8-days post infection. The X-axis represents the amount of bacteria in the spleen tissue and is expressed as log_10_ of the colony forming units per gram of tissue. The Y-axis represents the amount of bacteria in peripheral blood and is expressed as log_10_ of the bacterial genome copy number per 10µL of blood.

**Figure S2. Bacterial burden in peripheral blood among C, R and S chicks at 1-day post infection.** Data are presented as log_10_ of the bacterial genome copy number per 10µL of peripheral blood. Serovar-specific qPCR was used to quantify the *S. enteritidis* in blood, as described in Li et al. 2017. Six chicks in each group were selected. No *S. enteritidi*s was detected in the Controls. Each bar represents Mean ± SEM. ** indicate significant differences (*P* < 0.01) between S and R group. C = Controls (non-infected birds); R = Resistant birds; S = Susceptible birds.

**Table S1. Overview of raw data output and quality assessment.**

**Table S2. List of all differential expression of mRNAs in S vs. C group.**

**Table S3. List of all differential expression of mRNAs in R vs. C group.**

**Table S4. List of all differential expression of mRNAs in S vs. R group.**

**Table S5. qPCR primers used in this study.**
