# Supplementary figures and images for: Messenger RNA Sequencing and Pathway Analysis Provide Novel Insights Into the Susceptibility to Salmonella enteritidis Infection in Chickens
Source: Front Genet. 2018 Jul 13;9:256. doi: 10.3389/fgene.2018.00256 (PMC6055056; doi:10.3389/fgene.2018.00256)

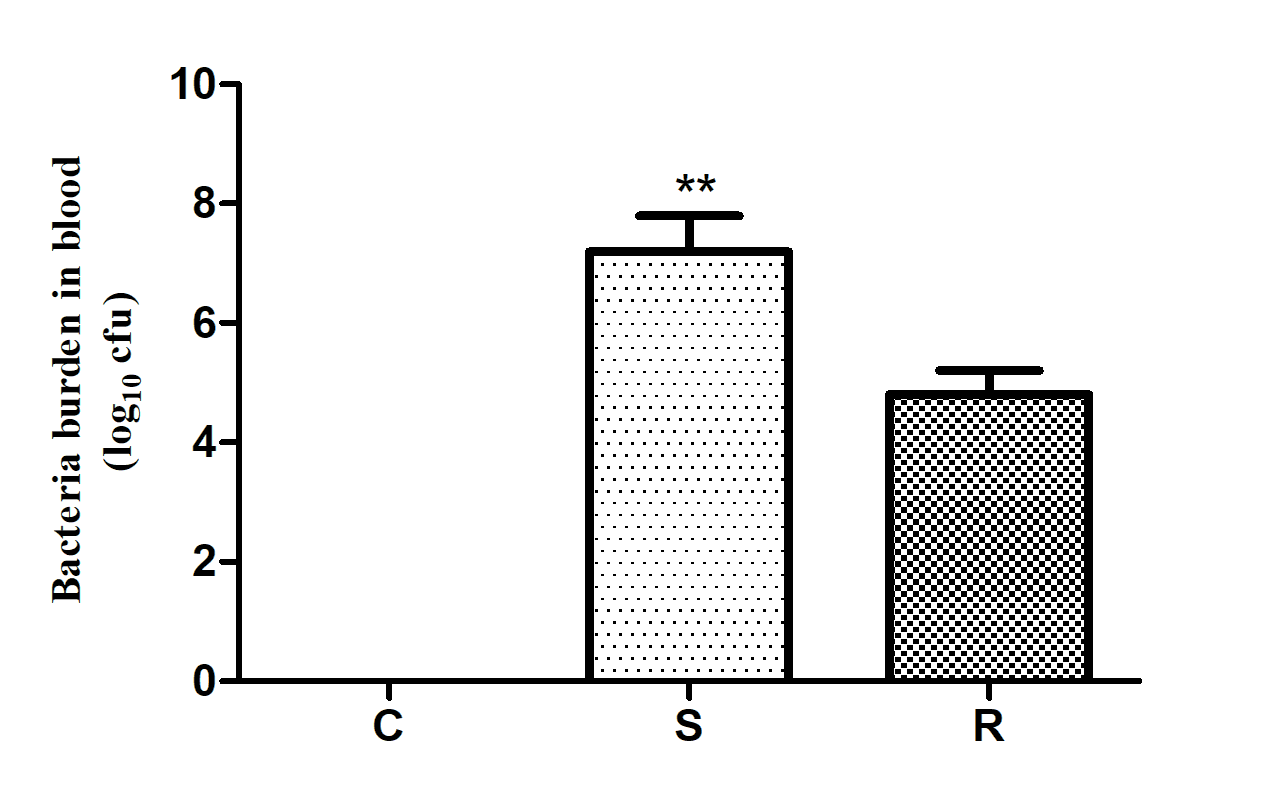

Supplement: Supplementary file 4 [file Image_2.TIF]
